# Study on essential drug use status and its influencing factors among cerebral infarction inpatients in county level hospitals of Anhui Province, China

**DOI:** 10.1371/journal.pone.0193513

**Published:** 2018-04-05

**Authors:** Cunhui Wang, Niannian Li, Heng Wang, Hongyan Yin, Yunwu Zhao

**Affiliations:** 1 Social Science and Health Management School, Anhui Medical University, Hefei, China; 2 Department of Research Administration, The First Affiliated Hospital of Anhui Medical University, Hefei, China; 3 Department of Deam’s Office, The First Affiliated Hospital of Anhui Medical University, Hefei, China; 4 Department of Human Resources, The First Affiliated Hospital of Anhui Medical University, Hefei, China; Karolinska Institutet, SWEDEN

## Abstract

**Background and purpose:**

Drug costs is one of the main components of hospitalization expenditure for cerebral infarction inpatients. In China, the National Essential Medicine System (NEMS) was created to relieve the heavy drug-cost burden for patients. The objective of this study was to investigate essential drug-use status and its influencing factors among cerebral infarction inpatients in county-level hospitals of Anhui province, China.

**Methods:**

Three county-level hospitals were selected through a multi-stage cluster random sampling method. The hospitalization cost data of cerebral infarction inpatients in the three hospitals were extracted from the Anhui provincial information platform of the New Rural Cooperative Insurance System (NCMS), and whether the proportion of essential drug cost in the total drug cost reached the median value of 33.05% which was set as the evaluation index for essential drug-use status. Questionnaires for hospitals and physicians were designed and given to them to assess influencing factors.

**Results:**

We retrieved the cost data of 2,189 inpatients from the NCMS platform and investigated 51 corresponding physicians in total. The drug costs accounted for 52.6% of the total hospitalization cost, and essential drug costs alone accounted for 37.0% of the total drug costs. The essential drug-cost proportion was high among physicians with a higher recognition degree on NEMS, older age, lower final academic degree, longer work experience and lower professional title. Married physicians and those with tight organizational affiliation also prescribed more essential drugs.

**Conclusions:**

Increasing the proportion of essential drugs was an effective way to reduce the disease burden for cerebral infarction patients. Perfecting the NEMS, increasing government investment, reinforcing education and propaganda, and formulating relevant incentive and restrictive mechanisms were all effective ways to promote and increase the number of essential drug prescriptions written by physicians.

## Introduction

Stroke is the leading cause of disability and the second leading cause of death worldwide [1]. Cerebral infarction, caused by blockage of an artery supplying blood to the brain [2], accounts for about three quarters of all stroke cases [3, 4]. It has the characteristics of high morbidity, high mortality and high recurrence, and has a trendency of striking younger people. This has already become one of the most severe global public health problem [5].

China, as the largest developing country worldwide, is facing the same intractable situation. The official statistics show that the prevalence rate of stroke was 0.4%, 0.58%, 0.66%, 0.97% and 1.23%, respectively in the years1993, 1998, 2003, 2008 and 2013, and the yearly growth rate was 5.8%. The death toll of patients with cerebrovascular disease was 1,880,000 annually in China, and the potential years of life lost rate (PYLLR) was 580.81/100,000 standardized by the world population [6]. An even serious problem is that the mean age of onset for Chinese population is 63 years old, 10 years younger than that of Americans. In addition, patients under 45 years old account for nearly 15% of all the cases [7]. This disease has already produced a heavy direct and indirect economic burden for the whole nation.

The inpatient care treatment cost of cerebral infarction was 6.8 billion US dollars in China, and the yearly growth rate of the hospitalization expense was 24.96% from 2004, after eliminating the influence of price factors [8]. As the phenomenon of “drug-maintaining- medicine” exists in China for a long time, hospitals make a significant proportion of their income for themselves and physicians via the drug procurement process[9]. Consequently, the drug cost accounted for nearly 60% of the total expense for cerebral infarction patients [5, 10–11], and this ration appreciably exceeded the international ratio of less than 15% [12]. It is critical now to seek ways to reduce the hospitalization expenses, especially the drug costs to ease the disease burden for this patient group in China.

Essential medicines, a concept proposed by the WHO, refers to those that satisfy the priority health-care needs of the population. They are selected with due regard to public health relevance, evidence on efficacy and safety, and comparative cost-effectiveness [13]. One of the goals for this policy was to reduce the rapid growth of medical cost globally. So far, this concept has produced considerable benefits worldwide [14–17]. In 2009, increasing concerns regarding the appropriateness of medical drug use and access to safe and effective essential medicine led the Chinese government to introduce the National Essential Medicine System (NEMS) for primary public healthcare facilities [18].After nearly seven years of practice, this system has already extended to county-level hospitals, which typically act as secondary-level general hospitals and play a pivotal role in the national health-care system. Further, in June 2012, China began a program of comprehensive reform of these hospitals that created suitable conditions for the implementation of the Essential Medicine System (EMS) [19].

Anhui is a province located in eastern China. It has a population of approximately 70 million, the majority of which are rural residents who are covered by the health insurance of the National New Rural Cooperative Medical System and who use the county-level hospitals as their first choice when needed. This province is one of the four health-reform pilot provinces; it has been at the forefront of the NEMS and county-level hospital reform and has created many innovative measures that are popularized to the whole nation.

Many relevant theoretic and empirical studies have been reported since the policy implementation, such as a cost-effectiveness analysis of essential medicine among inpatients, comparing the total cost change before and after the reform or evaluating the availability, prices, and affordability of essential medicines after the policy implementation [18, 20–24].However, there are no studies on the prevalence of essential drug use and its influencing factors among the inpatients of specific diseases. In view of the severity and high burden of cerebral infarction to society and the critical role of county-level hospitals in China, this study intended to investigate the essential drug-use status and its influencing factors among cerebral infarction inpatients from the perspective of institutions and physicians in the county-level hospitals of Anhui province.

## Methods

### Sample and data collection

A multistage cluster random sampling method was used in this study. First, we randomly selected three prefecture-level cities according to the geographical position of northern, middle, and southern Anhui, which are Huainan, Hefei, and Wuhu. Then, we randomly chose a county-level hospital from each of the aforementioned cities as the investigation scene (coded as A, B, and C, respectively).

The original questionnaires for the hospitals and physicians were designed based on a literature review [25–28] and expert consultation, and they were improved by examining participants’ understanding of the items and the content validity of the questionnaire after a pilot survey [29].

The questionnaire for hospitals included data on bed number, the ratio of doctors and nurses, the ratio of beds and nurses, daily inpatient care per doctor, the volume of medical services (outpatient/emergency man-times and inpatient man-times), general income, drug income, total expenditure (include drug expenditure), pharmacy income proportion, and the procurement proportion of essential medicines. All the above data was for the year 2015. The questionnaire for physicians was mainly to ascertain their degree of recognition on the NEMS. Ten items were investigated, including awareness, acceptance and support of the essential medicine list concept among patients, the propaganda effect, the education and training extent, the incentive and restraint mechanisms of hospitals, the government compensation degree, the credibility between doctors and patients, the accessibility of essential drugs, and the rationality of drug use. All the above items were divided into five levels using a Likert Scale, which were scored from 1 to 5. Physicians choose the corresponding score that they think best represents their beliefs to the particular item, and an average score for all the ten items together was calculated as the general recognition degree on the NEMS for those physicians. A higher score means a more positive attitude and acceptance of the NEMS. The formal field investigation was carried out from May 10–July 17, 2016. First, we extracted hospitalization costs data of cerebral infarction inpatients from the Anhui provincial information platform of the New Rural Cooperative Insurance System (NCMS).The search range was cerebral infarction inpatients that were discharged from January 1–December 31, 2015 from the three sample hospitals. Cerebral infarction was operationalized to include occlusion of cerebral arteries (International Classification of Disease, 9^th^ Revision (ICD9) code 434) and acute but ill-defined cerebrovascular disease (ICD-9) code 436) [30]. The extracted information included inpatients’ age, sex, length of stay (LOS), which was defined as the date of admission for cerebral infarction until the patient was discharged from the hospital to home [31], total cost, total drug cost and total cost for essential medicines. At the same time, the names of their charge physicians were recorded to request the corresponding physician to fill in the physician’s questionnaire at the next stage of field investigation. Questionnaires for the three hospitals were filled in by the responsible person respectively. All the questionnaires for both physicians and hospitals were checked for data integrity and logical errors by our investigators right after receipt. If there were any omission or error, the respondents were asked to correct it to ensure data validity.

All the research methods and investigational tools in this study were approved by the Ethics Committee of the First Affiliated Hospital of Anhui Medical University. All the respondents in this study were informed about the study objectives, procedures, and confidentialities via verbal informed consent. All the data were analyzed anonymously.

EpiData3.1 was used for data entry to establish the database and SPSS17.0 (SPSS Inc., Chicago, IL, USA) was used for statistical analysis. Descriptive analysis was used for hospital variables, physicians’ and patients’ demographics, physicians’ recognition scores for NEMS, and patients’ hospitalization cost. Finally, the three databases for hospitals, doctors, and patients were integrated into one to perform influence factor analysis. Multiple logistics analysis was used to conduct the multivariate analysis. As an indicator of the essential drug-use status, we used the proportion of essential drug cost in the total drug cost during the hospitalization period among cerebral infarction inpatients. For the proportion of essential drug cost in the total drug cost was skew, so we transformed this value into binary-classification, and the median value of 33.05% was used as the cutoff value. When the proportion of essential drug cost in the total drug cost is higher than 33.05%, this was regarded as a dependent variable. Hospitals, physician’s age, gender, final academic degree, marital status, work experience, professional title, monthly income, organizational affiliation, their mean score to the NEMS and patient’s age, gender and LOS were set as independent variables. For the small number of covariates, single factor analysis was omitted and all the above 13 independent variables were included in the multiple logistics analysis directly. The forward method was applied to introduce the variables into the regression equation, with the inclusion criteria α equal to 0.05 and the exclusion criteria α up to 0.1. First, dummy variables for the five multicategorical variables (physician’s final academic degree, work experience, professional title, monthly income, and organizational affiliation) were set. Then, the dummy variables of each dimension were introduced into the model as a whole and entered into the equation. The level of significance was set at P≤0.05.

## Results

In total, three hospitals and fifty-one physicians were investigated, and 2,189 inpatients’ cost data were obtained from the Anhui provincial information platform of the NCMS.

### General conditions of hospitals, doctors and inpatients’ costs

The general information and relevant business data of the three study hospitals are shown in Table 1.

**Table 1:** General and relevant business data of the three hospitals in 2015.

Variable	A	B	C
**Bed number**	440	426	479
**Ratio of doctors and nurses**	1/1.7	1/1.17	1/1.37
**Ratio of beds and nurses**	1/0.51	1/0.62	1/0.58
**Daily inpatient cases per doctor**	2.70	1.81	1.67
**Medical service volume(ten thousand)**	27.2	38.9	35.5
**General income (ten thousand USD)**	2186.3	2588.5	2122.2
Drug income (ten thousand USD,%)*	817.9(37.4)	910.2(35.2)	804.9(37.9)
**Total expenditure (ten thousand USD)**	2087.8	2434.8	2117.8
Drug expenditure (ten thousand USD,%)*	827.6(39.6)	912.1(37.5)	801.5(37.8)
**Procurement proportion of essential medicine (%)**	13.73	16.56	23.53

* %: the proportion of drug income (expenditure) in the general income (total expenditure).

In Table 2, it showed a total of 51 charge physicians (34 males and 17 females) completed the questionnaire with an average age of 36 years old (range: 24–59 years old). The other sociodemographic characteristics are also shown in Table 2.

**Table 2:** Sociodemographic characteristics of the 51 physicians.

Variable		Hospital A	Hospital B	Hospital C	Total (%)
**Gender**	Male	10	10	14	34(66.7)
	Female	4	8	5	17(33.4)
**Final academic degree**	Junior college and below	4	3	0	7(13.7)
	Bachelor’s degree	10	8	18	36(70.6)
	Master’s degree and above	0	7	1	8((15.7)
**Marital status**	Unmarried	1	1	4	6(11.8)
	Married	13	17	15	45(88.2)
**Work experience**	≤2 years	2	0	1	3(5.9)
	3–4 years	4	5	3	12(23.5)
	5–9 years	4	6	6	16(31.4)
	10–15 years	3	4	3	10(19.6)
	≥15 years	1	3	6	10(19.6)
**Professional title **	None	2	0	0	2(3.9)
	Primary	6	11	10	27(52.9)
	Intermediate	5	7	7	19(37.3)
	Senior	1	0	2	3(5.9)
**Monthly income(USD)**	≤5000	5	1	3	9(17.4)
	501–750	6	16	9	31(60.8)
	751–1000	3	1	5	9(17.6)
	≥ 1000	0	0	2	2(3.9)
**Organizational affiliation**	personnel on payroll	3	15	19	37(72.5)
	human agency	6	2	0	8(15.7)
	contract labor	5	1	0	6(11.8)

The average score of physicians’ recognition on the NEMS and its effect is 2.69 (Table 3). For the ten items separately, they agree that patients’ acceptance of essential drugs have improved most with a score of 2.94. However, the physicians are least satisfied with the insufficiency of government compensation, which only scored 2.24. The scores for the ten items are shown in Table 3.

**Table 3:** Physician’s recognition score on the supporting degree from patients, hospitals, government to the EMS and its effect.

Hospital	Patients’ awareness	Patients’ acceptance	Patients’ support degree	propaganda effect	education and training extent	incentive and restraint mechanism	government compensation	credibility between doctors and patients	accessibility	rationality of drug use	Mean
**A**	2.36	2.71	2.57	2.64	2.36	2.36	2.43	2.29	2.79	2.64	2.52
**B**	3.06	3.00	2.89	3.00	2.94	2.67	2.22	2.22	3.00	3.06	2.81
**C**	2.95	3.05	2.63	2.79	3.05	2.47	2.11	2.42	2.79	2.95	2.72
**Mean**	2.82	2.94	2.71	2.82	2.82	2.51	2.24	2.31	2.86	2.90	2.69

The 2,189 patients included 1,134males and 1,055 females with an average age of 70.9 years old. Their average LOS was 7.32 days, and the average total hospitalization cost was 631.8USD. The average cost for drugs and essential drugs were332.5USD and 123.0USD, respectively, and the average proportion of essential drug cost in the total drug cost was 37.0%. The specific hospitalization cost information for the 2,189 patients (classified by hospital) are shown in Table 4.

**Table 4:** Hospitalization cost (USD) data for the 2,189 cerebral infarction patients.

Hospital	Patient’s age	LOS	Total cost	Total drug cost (%)*	Cost for essential medicine	Proportion of essential drug cost in the total drug cost (%)
**A**	72.05	6.73	530.9	263.4(49.6)	135.2	51.3
**B**	69.69	8.32	754.3	358.8(47.6)	113.8	31.7
**C**	70.8	7.49	610.2	375.4(61.5)	119.9	31.9
**Mean**	70.87	7.5	631.8	332.5(52.6)	123.0	37.0

(%)* proportion of total drug cost in the total cost

### Multifactor analysis of the influencing factors on essential drug-cost proportion

The multifactor analysis result indicated that the difference between patient’s age and their gender, LOS as well as physician’s monthly income had no statistical significance to the essential drug-use status for the cerebral infarction patients.

The essential drug-cost proportion was higher for older physicians and those with a higher score on the NEMS. Physicians with a higher academic degree and a longer work experience tended to prescribe less essential drugs. The essential drug-cost proportion was much higher for physicians with no professional title than those with a senior title. Physicians who were personnel on the payroll or employed through a human agency prescribed less essential drugs than contract laborers. And the essential drug-cost proportion was much higher in hospital C than other two hospitals (see Table 5).

**Table 5:** Multifactor analysis of the elements that affected essential drug cost proportion.

Variable	B	S.E.	Wald	df	Sig.	Exp (B)	95% C.I.for EXP(B)	
							Lower	Upper
**Physician’s general recognition degree on EMS**	.288	.081	12.768	1	.000*	1.334	1.139	1.563
**Physician’s age**	.255	.030	72.954	1	.000*	1.290	1.217	1.367
**Physician’s gender**	-2.759	.202	186.647	1	.000*	.063	.043	.094
**Physician’s final academic degree**			139.209	2	.000*			
Junior college and below	6.348	.633	100.683	1	.000*	571.281	165.330	1.974E3
Bachelor’s degree	-.364	.278	1.712	1	.191	.695	.403	1.198
**Physician’s marriage status**	-1.023	.332	9.500	1	.002*	.360	.188	.689
**Physician’s work experience**			179.831	4	.000*			
≤2 years	12.300	1.208	103.589	1	.000*	2.197E5	2.056E4	2.347E6
3–4 years	4.890	.640	58.327	1	.000*	132.998	37.914	466.546
5–9 years	5.731	.540	112.721	1	.000*	308.234	107.009	887.854
10–15 years	2.920	.383	58.062	1	.000*	18.542	8.749	39.295
**Physician’s professional title**			99.645	3	.000*			
None	6.351	1.174	29.251	1	.000*	572.961	57.359	5.723E3
Primary	-2.114	.473	20.017	1	.000*	.121	.048	.305
Intermediate	-.531	.399	1.773	1	.183	.588	.269	1.285
**Physician’s Monthly income(USD)**			.186	3	.980			
≤500	23.302	3.755E3	.000	1	.995	1.318E10	.000	.
501–750	23.207	3.755E3	.000	1	.995	1.199E10	.000	.
751–1000	23.278	3.755E3	.000	1	.995	1.287E10	.000	.
**Physician’s Organizational affiliation**			23.927	2	.000*			
personnel on payroll	-2.795	.584	22.908	1	.000*	.061	.019	.192
human agency	-2.865	.606	22.359	1	.000*	.057	.017	.187
**Hospitals**	.288	.081	12.768	1	.000*	1.334	1.139	1.563
**Constant**	-30.214	3.755E3	.000	1	.994	.000		

*Significance

## Discussion

The hospitalization-cost data of 2,189 cerebral infarction patients who were admitted to three county-level hospitals in Anhui province, China, was analyzed in this study. The average LOS for cerebral infarction patients was 7.5 days, which was similar to a study in California (8.6 days), but much lower than other domestic results from Zhejiang province (12.71days) and Beijing (30 days) [32–33] and the results from six European countries, Japan, and Taiwan [34–36]. Because LOS is a strong indicator for the costs of treatment [34], the average hospitalization cost (631.8USD) for our study patients was much less than that of Zhejiang (1237.6USD) and Beijing (1435.0USD). The shorter LOS and lower total cost in our study may be due to the fact that our study participants were patients in county-level hospitals whose conditions were better than those study patients in Zhejiang and Beijing who were admitted to top three hospitals. In China, the top three hospitals mainly receive and treat patients with difficult and miscellaneous diseases whose treatment courses are much longer than for patients in county-level hospitals with milder clinical symptoms. Although compared to other studies, our patients spent less during their hospital stay, the disease burden was not so light with respect to their annual income. According to the report of the Anhui Provincial Statistics Bureau, the rural residents’ per capita disposable income in 2015 was only 1572.8USD [37]. A single hospitalization cost would account for almost half of their yearly income, and this has generated a heavy burden for those patients and their families. After carefully analyzing the cost composition, we found that the proportion of drug cost in the total cost was as high as 52.9%. This percentage was consistent with other domestic research, such as 52–56% reported by Y. Ma [10], 60.7% by Ding Hong [38], and 55% by XueliangZu[32]. These high percentages give us an indication that drug abuse may still exist in China. However, we gained a gratifying finding in the following analysis that the higher proportion of essential medicines in the total drug cost, the lower the drug cost and the total cost. Our finding verified other previous domestic studies that essential medicines are effective ways to reduce patients’ average drug expenditure and hospitalization costs [39]., without compromising care due to the robust methodology for developing EMLs[14–17].

Multifactor analysis showed that the essential drug-cost proportion was higher among physician**s** with a higher score on the NEMS. This result can be explained by the KAP model [40]. In other words, the more knowledge they have on essential medicine, the more positive attitude they hold toward it, which leads to less severe prescribing practices. Thus, increasing physicians’ overall recognition of essential drugs is critical for increasing their use of essential drugs. With regard to the ten items, physician**s** were least satisfied with government compensation, which was scored only 2.24. The essence of EMS is a “zero mark-up” policy, which means the profit from drug selling is eliminated, and this revenue previously accounted for more than 50% of hospital revenue in China[41]. In this case, hospitals will struggle to survive without sufficient investment [42]. Consequently, the government has the unshakable duty to establish a scientific, appropriate, and effective compensation mechanism to ensure the sustainable development both for hospitals and the NEMS itself. The second least-satisfied item for physician**s** was credibility between doctors and patients. In current Chinese society, hospital-patient conflicts occur frequently. The great majority of doctors have a passive attitude toward doctor-patient relationship and the overall medical environment. To protect themselves and avoid conflict, some physician**s** may prescribe nonessential medicines, which seem to have a quicker effect in inhibiting illness to cater to a patient’s illusion that expensive drugs are better than cheap ones. Improving the occupational environment and enhancing trust between doctors and patients are also essential for the priority selection of essential drugs among physician**s**. Enhancing public awareness and education and establishing effective communication channels are also feasible methods. The third least-satisfied item for physician**s** on the EMS was incentive and restraint mechanisms by hospitals. Because of an insufficient government subsidy, hospitals are assigned income-generating tasks without strict implementation of relevant reward and punishment [43]. The absence of incentive and restraint mechanisms from the hospital gives the physician**s** no incentive to choose essential medicines rather than other drugs with higher prices because they can obtain a sizeable commission from medical representatives for prescriptions of innovator brands [20]. A clear, strict, feasible, and efficient incentive and restraint system should be formulated to cut off the benefit chain between doctors and medical representatives and to improve physicians’ motivation to prescribe essential medicine.

Besides a physician’s recognition on the NEMS, the multifactor analysis also indicated that some sociodemographic characteristics are also influencing factors for their essential drug usage. Female physicians prescribe more essential drugs than males; this was different from Orzella’s study which demonstrated that female doctors prefer more drugs than male doctors [44]. In this study, physician**s** with a higher academic degree or a senior professional title tended to prescribe less essential drugs for cerebral infarction patients. Research by Wang Heng, et al. also found that doctors with higher degrees tended to prescribe more drugs and fewer essential medicines [45]. This phenomenon reminds us that arrogance may exist among highly educated doctors who mistakenly believe that they know so much that they do not have to be bound by the rules of the NEMS. The essential drug-cost proportion was lower for physician**s** with a work experience of more than 15 years. This result was in accordance with Andersen et al., who indicated that long-time work experience would actuate doctors to follow their usual habits rather than opting for the best drugs [46]. Finally, physician**s** who are personnel on the payroll or contracted through a human resources agency prescribed less essential drugs than contract laborers. This is probably because in China, contract laborers has a loose tie with its organization and they are urgent to change their status into the other two personnel types under the premise of complying with every medical policy and do a good job in their work.

In addition, compared to the hospital in Wuhu, the essential drug-cost proportion in the total drug cost was lower in the other two hospitals. This was due to the fact that Wuhu was the first city in Anhui that initiated the EMS, so the policy effect was stronger. However, we have noted that the procurement proportion of essential medicine in the three hospitals was far below the provincial requirement of no less than 50% [47]. This insufficient procurement proportion of essential medicine inevitably reduced the essential drug-use proportion in the total drugs used for cerebral infarction patients. This deficiency may be the result of the following two reasons. First, the National Essential Medicines List was not appropriate for every county-level hospital because of the different medical demands in each hospital’s service radius. Supplement or adjustment of the list on the basis of the specific common and frequent disease scope at the provincial level may be necessary to give both hospitals and physician**s** more drug-use options. Secondly, the result of the regulations of medicine bidding and procurement files, which state that hospitals can only chose the brand with the lowest price from the winning manufacturers. However, certain manufacturers ceased production of some essential drugs after winning the bid because the low price and strict procurement procedures severely curtailed their profit, and this directly caused the hospitals had no access to the necessary essential medicines [24].

Drug costs currently account for the largest amount of the total hospitalization expense for cerebral infarction patients in China. Increasing the proportion of essential drugs is an effective way to reduce the disease burden for this patient group. However, how to promote essential drug prescriptions by physician**s** is a complex issue that is not only related to the doctors themselves. Perfection of the NEMS, an increase in government investment, reinforcement of education and propaganda for physician**s**, the formulation of relevant incentives and restrictive mechanisms, and other measures are necessary to ensure this happens and ease the financial burden for patients without compromising care.

## Supporting information

S1 File Study data. (XLS)
